# STAC3 stably interacts through its C1 domain with Ca_V_1.1 in skeletal muscle triads

**DOI:** 10.1038/srep41003

**Published:** 2017-01-23

**Authors:** Marta Campiglio, Bernhard E. Flucher

**Affiliations:** 1 Department of Physiology and Medical Physics, Medical University of Innsbruck, 6020 Innsbruck, Austria

## Abstract

The adaptor protein STAC3 is essential for skeletal muscle excitation-contraction (EC) coupling and a mutation in the *STAC3* gene has been linked to a severe muscle disease, Native American myopathy (NAM). However the function of STAC3, its interaction partner, and the mode of interaction within the EC-coupling complex remained elusive. Here we demonstrate that STAC3 forms a stable interaction with the voltage-sensor of EC-coupling, Ca_V_1.1, and that this interaction depends on a hitherto unidentified protein-protein binding pocket in the C1 domain of STAC3. While the NAM mutation does not affect the stability of the STAC3-Ca_V_1.1 interaction, mutation of two crucial residues in the C1 binding pocket increases the turnover of STAC3 in skeletal muscle triads. Thus, the C1 domain of STAC3 is responsible for its stable incorporation into the Ca_V_1.1 complex, whereas the SH3 domain containing the NAM mutation site may be involved in low-affinity functional interactions in EC-coupling.

Excitation-contraction (EC) coupling is a fundamental process of muscle physiology, in which an electrical signal, the action potential, is converted into a mechanical response, muscle contraction. In skeletal muscle transverse (T−) tubules, membrane depolarization is sensed by the voltage-gated calcium channel Ca_V_1.1 (also named dihydropyridine receptor; DHPR) which activates calcium release from the sarcoplasmic reticulum (SR) via protein-protein interactions with the type 1 ryanodine receptor (RyR1). The resulting elevation of the cytoplasmic calcium concentration triggers muscle contraction.

Consistent with their respective functions as voltage-sensor and calcium release channel, the Ca_V_1.1 α_1S_ and β_1a_ subunits^1^,^2^ and the RyR1^3^ were shown to be essential for skeletal muscle EC coupling. Recent knockout studies in mouse and zebrafish^4^,^5^ revealed the essential role in EC coupling of a hitherto unnoticed muscle protein, STAC3, which belongs to the family of SH3 and cysteine-rich containing adaptor proteins with three members (STAC1, STAC2, and STAC3). Expression of STAC3 is specific to skeletal muscle, whereas STAC1 and STAC2 are expressed in brain and a variety of other tissues, but not in skeletal muscle^4^. In both fish and mouse, knockout of STAC3 resulted in paralysis and perinatal death from suffocation, similar to the phenotype of the other EC coupling null models^4^,^5^. Both knockout models revealed also that STAC3 is required for voltage-dependent calcium release from the SR. However, the primary interaction partner of STAC3 in the triad and the molecular mechanism of its function in EC coupling remain elusive. The importance of STAC3 for EC coupling is further supported by a point mutation in the human *STAC3* gene that has been linked to congenital Native American myopathy (NAM), which is characterized by multiple clinical features including muscle weakness and susceptibility to malignant hyperthermia^5^,^6^.

More recently, Polster *et al*.^7^ demonstrated that STAC3 is required for efficient membrane trafficking of Ca_V_1.1 in heterologous cells. In fact Ca_V_1.1 is the only voltage-gated calcium channel that expresses poorly in non-muscle mammalian cells. However, co-expression of STAC3 promoted robust functional membrane expression of Ca_V_1.1 in tsA201 cells^7^. This suggested that impaired Ca_V_1.1 membrane trafficking might also cause the EC coupling deficiency in the STAC3 knockout models^4^,^5^. However, EC coupling was still impaired in muscle fibers of STAC3-null zebrafish embryos 48 hours post-fertilization, which contained normal levels of Ca_V_1.1^5^ and in STAC3 KO mouse myotubes, which contained substantial, but reduced, amount of Ca_V_1.1^8^. Thus, STAC3 appears to perform multiple functions in Ca_V_1.1 trafficking and EC coupling.

Here, we examined the questions as to how STAC3 interacts with Ca_V_1.1 in skeletal muscle triads and whether this interaction is altered by the NAM mutation. Applying co-expression of different combinations of GFP-tagged STAC paralogs with Ca_V_1.1 or Ca_V_1.2 in dysgenic myotubes we demonstrate that in skeletal muscle triads STAC3 forms a stable complex with Ca_V_1.1, that this association is specific for STAC3 but not for Ca_V_1.1, and that the mutation causing NAM does not abolish this interaction. By generating multiple STAC2/STAC3 chimeras and point mutants we identified two residues in the C1 domain of STAC3 which are critical for its stable association with Ca_V_1.1 and Ca_V_1.2 calcium channels in skeletal muscle triads.

## Results

### STAC3 is stably incorporated in the triad junctions of dysgenic myotubes

**Figure 1:**
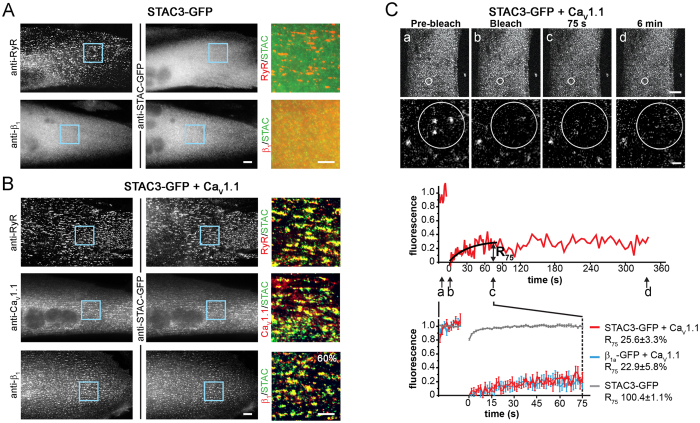
STAC3 is a stable component of the DHPR complex in skeletal muscle myotubes. (**A**) In dysgenic (Ca_V_1.1^−/−^) myotubes the RyR1 is distributed in discrete clusters in the triads, while STAC3-GFP and the endogenous β_1a_ are diffusely distributed in the cytosol. (**B**) On co-expression with Ca_V_1.1, STAC3-GFP is colocalized in clusters with RyR1 and the DHPR subunits Ca_V_1.1 and β_1a_ (lower panel; N = 4, n = 120). Color overlay: 4X of area marked with blue rectangle. Scale bars: 10 μm and 5 μm. (**C**) Clusters of STAC3-GFP/Ca_V_1.1 in live dysgenic myotubes were photobleached (within circles) and imaged up to 6 minutes. Representative high magnification images and the corresponding normalized FRAP recording (below) show little fluorescence recovery of STAC3-GFP in the bleached clusters (a–d, time points of example images; scale bars: 10 μm and 1 μm). The value of the fitted curve at 75 s after bleaching was used to calculate the fractional fluorescence recovery (R_75_). Average recovery curves (lower panel) reveal similar low fluorescence recovery of STAC3-GFP (red) and β_1a_-GFP (blue) in the presence of Ca_V_1.1 and comparable recovery rates 75 s after bleaching (R_75_) (mean ± s.e., N = 3, n = 13; Student’s t-test, P = 0.34)., The fluorescence of STAC3-GFP in the absence of Ca_V_1.1 recovers completely in few seconds (grey, from Fig. S1).

Because STAC3 is an essential component of skeletal muscle EC coupling, but its association with the voltage sensor Ca_V_1.1 and/or the RyR1 is controversial^5^,^7^, we first expressed STAC3-GFP in dysgenic (Ca_V_1.1^−/−^) myotubes. These muscle cells form junctions between the sarcoplasmic reticulum (SR) and the plasma membrane or T-tubules, which for reasons of simplicity we will subsume under the term triad. As shown in Fig. 1A triads in dysgenic myotubes contain RyR1 clusters but lack Ca_V_1.1. Consequently the endogenous Ca_V_β_1a_ subunit, which binds to Ca_V_1.1, is diffusely distributed in the cytoplasm. Similarly, in the absence of Ca_V_1.1 also heterologously expressed STAC3-GFP fails to colocalize with the RyR1 clusters and is diffusely distributed throughout the myotubes (Fig. 1A). However, when dysgenic myotubes were reconstituted with Ca_V_1.1, STAC3-GFP formed clusters and these STAC3 clusters were colocalized with Ca_V_1.1, β_1a_, and the RyR1 (Fig. 1B). Thus, upon reconstitution of dysgenic myotubes with Ca_V_1.1, both the β_1a_ subunit and STAC3-GFP redistributed from the cytoplasm to the triads. These results are consistent with similar previous observations^5^,^7^ and conclusively demonstrate that heterologously expressed STAC3-GFP incorporates in skeletal muscle triads only in the presence of Ca_V_1.1. For quantifying STAC3-GFP incorporation into triads we used double labeling with the β_1a_ subunit, because this would allow us to use the same antibody combination for the analysis of STAC3 co-clustering with Ca_V_1.1 and with Ca_V_1.2 (see below). 60.2 ± 6.1% of the transfected differentiated myotubes showed co-clustering of STAC3 with Ca_V_1.1.

The three previously identified essential core components of skeletal muscle EC coupling—Ca_V_1.1, β_1a_, and RyR1—form a stable complex with one another in the triad junctions of reconstituted dysgenic myotubes^9^. Because STAC3-GFP also partitions in the triad in a Ca_V_1.1-dependent manner and is essential for EC coupling^4^,^5^, we next examined whether it is a stable component of the EC coupling complex or a dynamically interacting modulator. In order to determine its dynamics in the triad complex, we applied a recently developed FRAP approach^9^. This method determines the fluorescence recovery after photobleaching specifically in the triadic clusters and compares the recovery rate and fractional recovery with that of other DHPR subunits. Similar recovery rates are indicative of a stable complex, the components of which turnover together at a slow rate. Higher recovery rates relative to Ca_V_1.1 indicate dynamic exchange of the respective protein with the calcium channel complex. The representative images of a ROI before and after photobleaching (Fig. 1C) and the corresponding recovery curve (below) show that, in the presence of Ca_V_1.1, STAC3-GFP fluorescence in the clusters does not substantially recover within the 6 min recording. Comparison of the average recovery curve of STAC3-GFP with that of β_1a_-GFP shows that that the dynamic properties of the two triad proteins are almost identical. The fractional fluorescence recovery value at 75 s (R_75_) of STAC3-GFP was 25.6 ± 3.3% of the pre-bleaching intensity and not significantly different from that of β_1a_-GFP (22.9 ± 5.8%), or the previously published values for Ca_V_1.1^9^. Together these results suggest that, in skeletal muscle triads, STAC3 is a stable component of the DHPR complex, similar to the β_1a_ subunit. Note that in the absence of Ca_V_1.1 the fluorescence of STAC3-GFP within the ROIs recovered at a much faster rate (Fig. 1C, gray trace; Fig. S1), as previously observed for soluble β_1a_-GFP^9^ and consistent with the notion that the DHPR complex is responsible for immobilizing STAC3 in the triads.

### The mutation causing Native American myopathy (NAM) causes reduced incorporation but normal stability of STAC3 in the calcium channel complex

**Figure 2:**
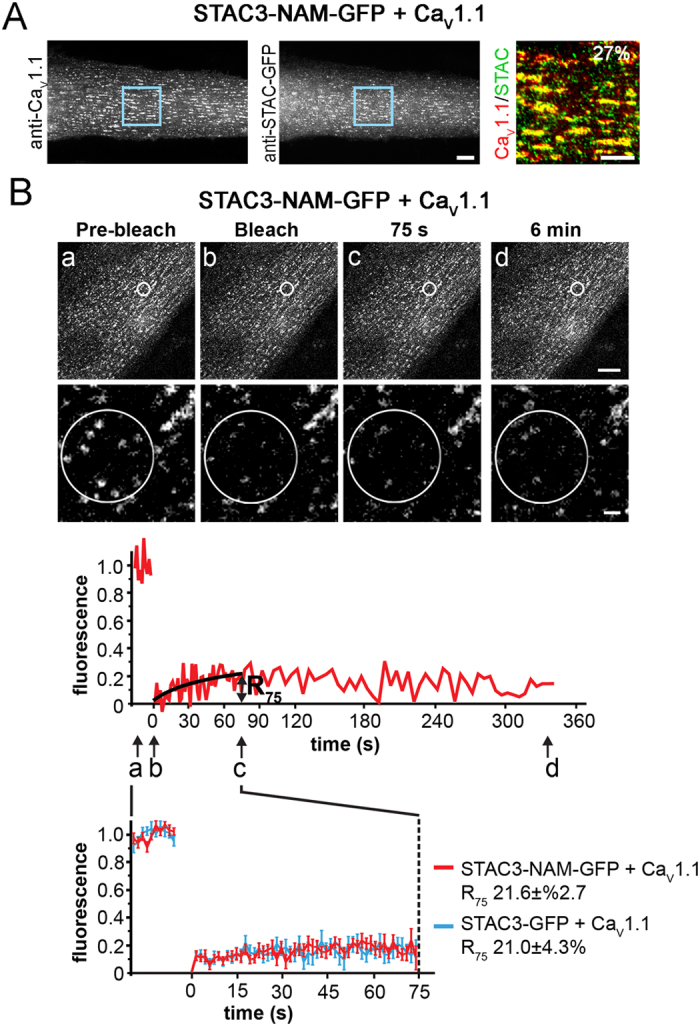
The NAM mutation does not affect the stability of STAC3 in the DHPR complex. (**A**) STAC3-NAM-GFP colocalizes in clusters with Ca_V_1.1, albeit at a reduced extent compared to STAC3-GFP (Fig. 1B) (26.9 ± 0.1%, N = 3, n = 80; P = 0.001). Color overlay: 4X of blue rectangle. Scale bars: 10 μm and 5 μm. (**B**) Fluorescence of STAC3-NAM-GFP co-expressed with Ca_V_1.1 did not recover within 6 min after bleaching. The mean FRAP curve and the R_75_ of STAC3-NAM-GFP were similar to that of STAC3-GFP (mean ± s.e., N = 3, n = 17; Student’s t-test, P = 0.46), suggesting that the NAM mutation does not alter the stability of STAC3 within DHPR complex. Scale bars: 10 μm and 1 μm.

The point mutation responsible for NAM substitutes a tryptophan with a serine in the first SH3 domain of STAC3^5^. When the mutated STAC3-NAM had been expressed in STAC3-knockout zebrafish myotubes, EC coupling was diminished, and localization of STAC3-NAM in the triads of the myotubes was reduced^5^. We therefore explored the possibility that the NAM mutation impaired the association of STAC3 with Ca_V_1.1 or that it affected its stability in the calcium channel complex. Upon co-expression in dysgenic myotubes STAC3-NAM-GFP colocalized with Ca_V_1.1 clusters (Fig. 2A). However, compared to wildtype STAC3 co-clustering of STAC3-NAM with the Ca_V_β_1a_ subunit was significantly reduced (STAC3-NAM, 26.9 ± 0.0.1%; STAC3, 60.2 ± 6.1%) and this was not due to differences in expression levels (Fig. S2). To examine whether the reduced co-clustering of STAC3-NAM resulted from its reduced stability within the DHPR complex, we analyzed the dynamics of STAC3-NAM in the triads using our FRAP protocol. After photobleaching STAC3-NAM-GFP in Ca_V_1.1 clusters showed little fluorescence recovery (Fig. 2B). Its mean recovery curve was practically identical to that of wildtype STAC3-GFP and R_75_ was not significantly different from that of STAC3 (STAC3-NAM 21.6 ± 2.7%; STAC3 21.0 ± 4.3%). The observation that the NAM mutation reduces STAC3 triad targeting but not the stability of STAC3 in the calcium channel complex suggests that the first SH3 domain of STAC3 may not be essential for its association with Ca_V_1.1, but rather for the Ca_V_1.1 targeting function of STAC3 or for its functional interactions within the EC coupling apparatus.

### STAC3 stably interacts also with the cardiac/neuronal Ca_V_1.2 expressed in triads of dysgenic myotubes

**Figure 3:**
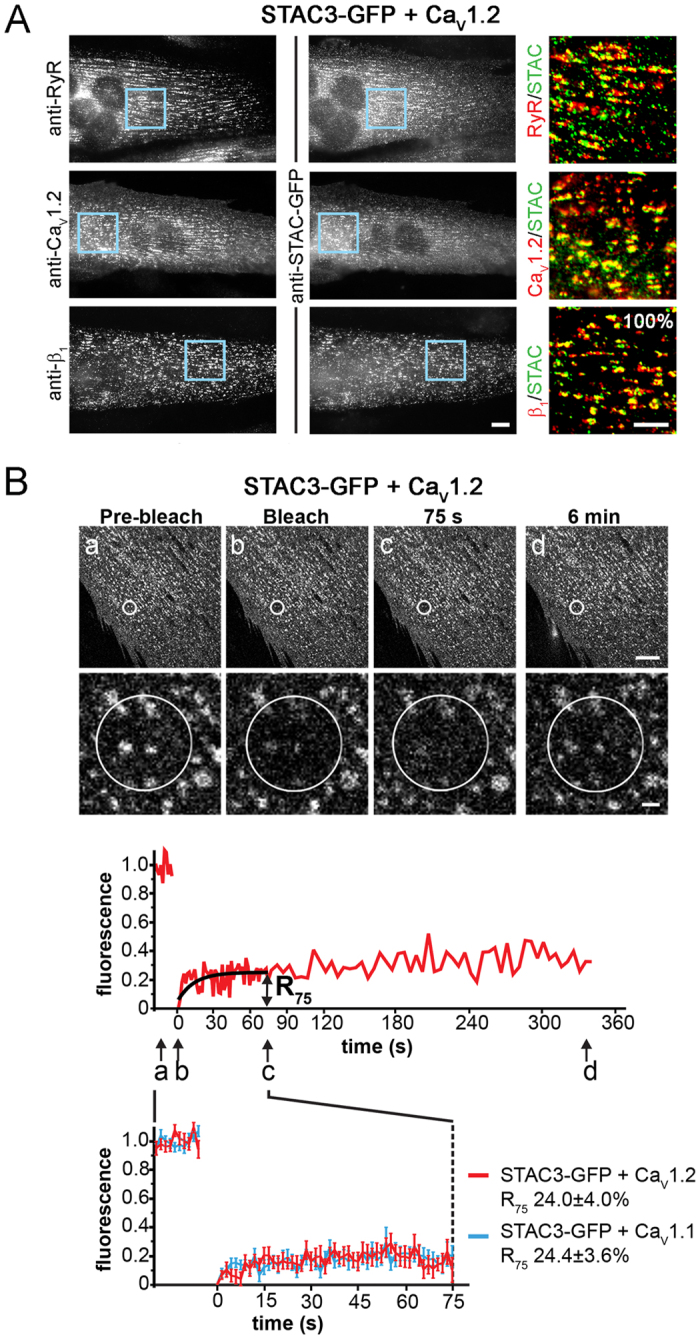
STAC3 colocalizes also with heterologous Ca_V_1.2 in skeletal muscle triads. (**A**) When co-expressed with Ca_V_1.2, STAC3 is colocalized in clusters with the RyR and the Ca_V_ subunits Ca_V_1.2 and β_1a_ in dysgenic myotubes. Color overlay: 4X magnification of blue rectangle. Scale bars: 10 μm and 5 μm. (**B**) FRAP analysis of STAC3-GFP co-expressed with Ca_V_1.2 shows very little recovery of fluorescence, very similar to when STAC3 is co-expressed with Ca_V_1.1. Average recovery curves reveal similar low fluorescence recovery of STAC3-GFP co-expressed with Ca_V_1.2 (red) and with Ca_V_1.1 (blue) and comparable recovery rates 75 s after bleaching (R_75_) (mean ± s.e., N = 4, n = 17, Student’s t-test, P = 0.47). Upper scale bar: 10 μm. Lower scale bar: 1 μm.

Polster and colleagues reported that, upon co-expression in tsA201 cells, STAC3 and STAC2 modulated the currents of the cardiac/neuronal Ca_V_1.2 channel^7^, suggesting promiscuous functional interaction between STAC proteins and L-type calcium channels in heterologous expression systems. We previously demonstrated that, when expressed in dysgenic myotubes, Ca_V_1.2 is incorporated into triad junctions and restored cardiac-type EC coupling^10^. Therefore we tested whether the cardiac/neuronal Ca_V_1.2 also recruits STAC3 into skeletal muscle triads and if so, whether their association is equally stable as that of the native partners STAC3 and Ca_V_1.1. When co-expressed in dysgenic myotubes STAC3-GFP and Ca_V_1.2 were colocalized in triad clusters (Fig. 3A). As expected, these STAC3 clusters were also colocalized the RyR1 and β_1a_ in dysgenic myotubes, confirming that the clusters represented triad junctions. Thus, the interaction with STAC3 is conserved between Ca_V_1.1 and Ca_V_1.2. Interestingly, quantitatively the ability of Ca_V_1.2 to recruit STAC3 into the DHPR complexes was even higher (100.0 ± 0.0%) than that of Ca_V_1.1 (60.2 ± 6.1%, Figs 1B and 3A). To assess the strength of association we measured the dynamics of STAC3 in Ca_V_1.2-containing DHPR complexes with FRAP. Again, very little recovery of fluorescence was observed. The mean recovery curve of STAC3-GFP in Ca_V_1.2 complexes was indistinguishable from that of STAC3-GFP in Ca_V_1.1 complexes (Fig. 3B), and also their R_75_ values were not significantly different (STAC3/Ca_V_1.2, 24.0 ± 4.0%; STAC3/Ca_V_1.1, 24.4 ± 3.6%). Together these results demonstrate that also the cardiac/neuronal Ca_V_1.2 very efficiently recruits STAC3-GFP to skeletal muscle calcium channel complexes and that the two heterologous protein partners associate with each other as stably as the native partners STAC3 and Ca_V_1.1.

### The three STAC isoforms differ in their ability to associate with Ca_V_1.1 and Ca_V_1.2 in skeletal muscle triads

**Figure 4:**
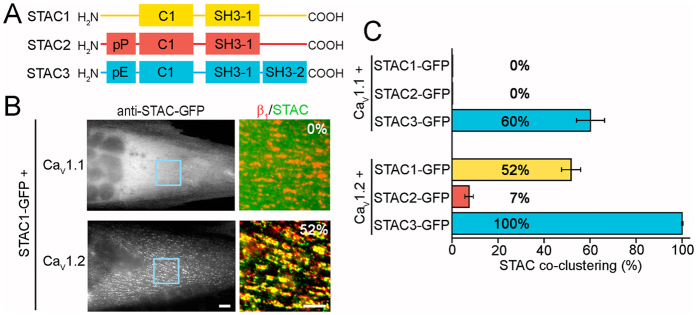
STAC1 and STAC2 display different degrees of colocalization with Ca_V_1.1 and Ca_V_1.2 in skeletal myotubes. (**A**) Domain structures of STAC1, STAC2, and STAC3. (**B**) STAC1 fails to show triad targeting in dysgenic myotubes expressing Ca_V_1.1, but co-clusters with the channel in about half of the myotubes expressing Ca_V_1.2 (51.8 ± 4.2%, N = 4, n = 120). Color overlay: 4X of blue rectangle. Scale bars: 10 μm and 5 μm. (**C**) Only STAC3 co-clusters with Ca_V_1.1, while the three STAC proteins display different degrees of co-clustering with Ca_V_1.2 (mean ± s.e.; N = 4, n = 120).

STAC proteins form a family of three isoforms: STAC1, STAC2, and STAC3. They all share a conserved protein kinase C (PKC) C1 domain and a src homology 3 (SH3) protein interaction domain (Fig. 4A). In addition STAC3 contains an N-terminal poly-E region (11 consecutive glutamic acid residues) and a second SH3 domain in the C-terminus. Instead STAC2 contains a poly-P region (proline rich) at its N-terminus and, like STAC1, lacks the second SH3 domain. STAC3 is the only STAC protein expressed in skeletal muscle and its expression is restricted to this tissue^4^. STAC1 and STAC2 are expressed in brain and a variety of other tissues, but their subcellular localizations or functions are not known^4^. In order to examine whether STAC1 and STAC2 also associate with L-type calcium channels, we analyzed their subcellular distribution in dysgenic myotubes reconstituted either with Ca_V_1.1 or with Ca_V_1.2. When expressed without a Ca_V_1 subunit, both STAC1-GFP and STAC2-GFP were diffusely localized in the cytoplasm of the myotubes (Fig. S3). In contrast to STAC3-GFP, this cytoplasmic distribution did not change when STAC1-GFP or STAC2-GFP were co-expressed with Ca_V_1.1 (Figs 4B,C and S3). This demonstrates that exclusively the skeletal muscle STAC3 isoform associates with the skeletal muscle Ca_V_1.1 in triads of dysgenic myotubes (Fig. 4C). This property does not result from different expression levels of the STAC paralogs (Fig. S2). Interestingly, when co-expressed with Ca_V_1.2, STAC1-GFP colocalized with Ca_V_1.2 clusters in approximately half of the myotubes (51.8 ± 4.2%, Fig. 4B), whereas co-clustering of STAC2-GFP and Ca_V_1.2 was only found in a small minority of differentiated myotubes (up to 7.4 ± 1.9% in one of four experiments; Figs 4C and S3). Together these findings indicate that STAC3 is the unique interaction partner of skeletal muscle Ca_V_1.1 and that both STAC1 and STAC2 might associate with and possibly modulate other voltage-gated calcium channels in their native tissues. In fact, a recent report suggests that STAC1 associates with T-type channels and promotes their functional expression in tsA-201 cells^11^.

### The C1 domain of STAC3 is necessary to confer to STAC2 the ability to associate with L-type calcium channels

**Figure 5:**
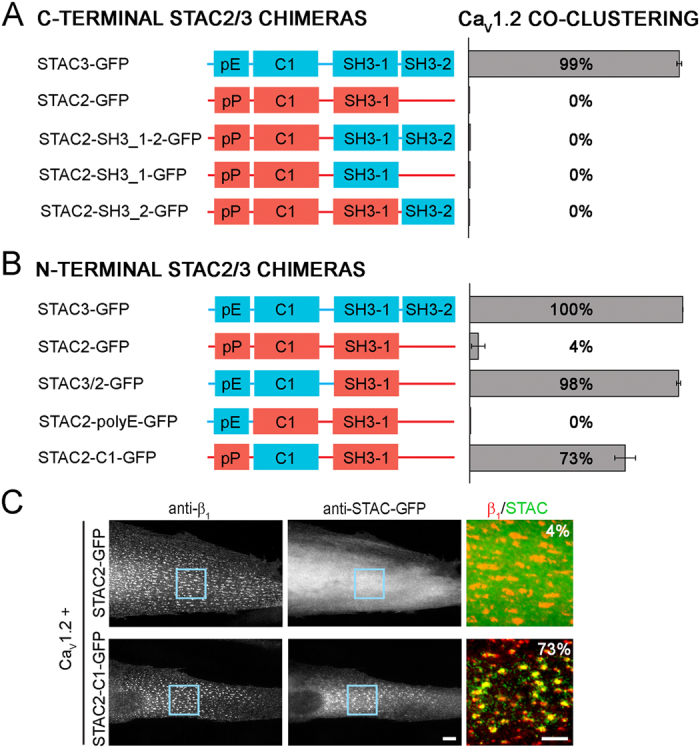
The C1 domain of STAC3 is necessary for the colocalization with L-type calcium channels. (**A**,**B**) Domain structure of the STAC2 and STAC3 isoforms and chimeras with STAC2 sequences in red and STAC3 sequences in blue. The bar graphs on the right show the percentage of transfected myotubes in which the expressed STAC isoform/chimera co-clustered with Ca_V_1.2/β_1a_. The two C-terminal SH3 domains (**A**; N = 3, n = 90) or the N-terminal pE and C1 domains (**B**; N = 4, n = 120) of STAC3 were inserted in the corresponding STAC2 sequences individually and jointly. (**C**) STAC2 showed triad targeting in none of the myotubes expressing Ca_V_1.2 but insertion of the C1 domain of STAC3 conferred to STAC2 triad targeting in about three fourths of the myotubes (for complete set of micrographs see Fig S3). Color overlay: 4X of blue rectangle. Scale bars: 10 μm and 5 μm.

The greatly different capacities of STAC2-GFP and STAC3-GFP to associate with Ca_V_1.2 enabled us to use a gain-of-function chimera approach to identify the sequence domain responsible for binding to the DHPR complex. To this end we systematically replaced STAC2 domains with the corresponding sequences of STAC3 and quantified the ability of the resulting chimeras to co-cluster with Ca_V_1.2 when co-expressed in dysgenic myotubes. Because the SH3 domains are well described protein interaction modules, and the NAM mutation, which reduced triad targeting, is located in the first SH3 domain, we first constructed three chimeras in which the SH3 domains of STAC3 individually or together were replaced with those of STAC2 (Figs 5A and S4A). Neither replacing the first or the second SH3 domains individually, nor replacing the entire C-terminus (containing both SH3 domains) conferred any degree of co-clustering with Ca_V_1.2 to the resulting chimera. Since the C-terminal chimeras did not prove successful in conferring Ca_V_1.2 co-clustering to STAC2, we continued substituting the N-terminus of STAC3 with that of STAC2 (Figs 5B and S4B). This chimera (STAC3/2-GFP) colocalized with Ca_V_1.2 clusters in almost all transfected myotubes (98.3 ± 1.0%). To determine which N-terminal region is responsible for this interaction, the poly-E and C1 domains of STAC3 were individually inserted in the corresponding regions of STAC2. Substitution of the poly-P domain of STAC2 with the poly-E domain of STAC3 (STAC2-polyE-GFP) did not improve triad targeting (0.0 ± 0.0%). In contrast, the substitution of the C1 domain of STAC2 with that of STAC3 (STAC2-C1-GFP) restored co-clustering of the chimera with Ca_V_1.2 in the large majority of myotubes (73.3 ± 4.9%) (Fig. 5B,C). This gain-of-function of the STAC2-C1-GFP chimera identifies the C1 domain of STAC3 as the protein sequence critical for the interaction of STAC3 with Ca_V_1.2.

### Amino acid residues V104 and Y133 of the C1 domain are crucial for the interaction of STAC3 with L-type calcium channels

**Figure 6:**
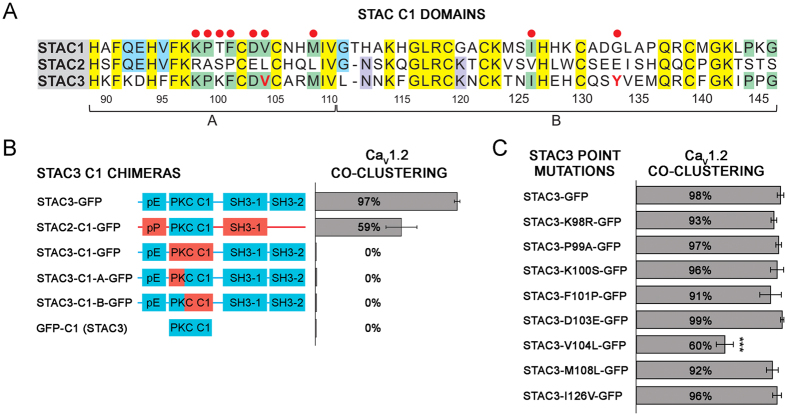
Residue V104 of STAC3 is important for its association with L-type calcium channels in triads of dysgenic myotubes. (**A**) Amino acid sequences of the C1 domain of all three mouse STAC proteins: Yellow, residues conserved in all three paralogs; green, conserved between STAC1 and STAC3; blue, conserved between STAC1 and STAC2; purple, conserved between STAC2 and STAC3. Red dots indicate STAC3 residues mutated to the corresponding residues of STAC2 (here and in Fig. 7). Numbers indicate the amino acid position of STAC3. (**B**) Domain structure of the STAC isoforms and chimeras with STAC2 sequences in red and STAC3 sequences in blue. Bar graphs on the right give the percentage of transfected myotubes in which the expressed STAC isoform/chimera co-clustered with Ca_V_1.2/β_1a_. Entire or partial substitution (indicated A and B in panel A) of the C1 domain of STAC3 by the corresponding sequences of STAC2 caused a total failure to colocalize with Ca_V_1.2. The isolated C1 domain of STAC3 was also not able to colocalize with Ca_V_1.2. (**C**) Single amino acid residues of STAC3 were systematically mutated to the corresponding residues of STAC2. Of these only STAC3-V104L-GFP resulted in a significantly reduced colocalization with Ca_V_1.2 (60.0 ± 5.8%). Anova F(10,22) = 44.8; P < 0.001 (P in the figure is for post-hoc analyisis; ***P < 0.001; N = 3, n = 90).

The C1 domains, also termed cysteine-rich or zinc finger domains, were originally described as lipid binding modules in protein kinase C. They consist of approximately 50 amino acids and contain the characteristic motif HX_12_CX_2_CX_n_CX_2_CX_4_HX_2_CX_7_C. The C1 domain is conserved in all three STAC paralogs (Fig. 6A), however in STAC3 two of the four crucial residues (G111L, Q115F) involved in binding phorbol ester differ^12^,^13^, suggesting an alternative function of the C1 domain in STAC3.

To further dissect its mechanism in the interaction with Ca_V_1.2, we constructed three chimeras in which the C1 domain of STAC3 was either entirely or partially replaced by that of STAC2 (Fig. 6B). As expected, when the entire C1 domain of STAC3 was replaced (STAC3-C1-GFP), the chimera lost its ability to colocalize in clusters with Ca_V_1.2 (0.0 ± 0.0%). Interestingly, also replacing only the first 22 (STAC3-C1-A-GFP) or the last 36 (STAC3-C1-B-GFP) amino acids with those of STAC2 resulted in a complete loss of Ca_V_1.2 association (both 0. 0 ± 0.0%). This suggested that residues from both the N-terminal and the C-terminal portion of the C1 domain directly or indirectly participate in the interaction with the calcium channel. In contrast, the isolated C1 domain of STAC3 (GFP-C1) by itself was not able to colocalize in clusters with Ca_V_1.2 (0.0 ± 0.0%), suggesting that the C1 domain of STAC3 requires other regions of STAC proteins to acquire the conformation necessary to engage in the interaction with the DHPR.

Because the ability to colocalize with Ca_V_1.2 was almost complete in STAC3, almost null in STAC2, and intermediate in STAC1, we generated STAC3 point mutants, in which the residues identical in STAC3 and STAC1 (highlighted in green in Fig. 6A) were mutated to the corresponding residues found in STAC2 (Fig. 6C). When we analyzed the ability of these point mutants to co-cluster with Ca_V_1.2, only a single mutant, STAC3-V104L-GFP, showed significantly reduced co-clustering with Ca_V_1.2 compared to STAC3-GFP (60.0 ± 5.8% and 97.8 ± 2.2%, respectively).

**Figure 7:**
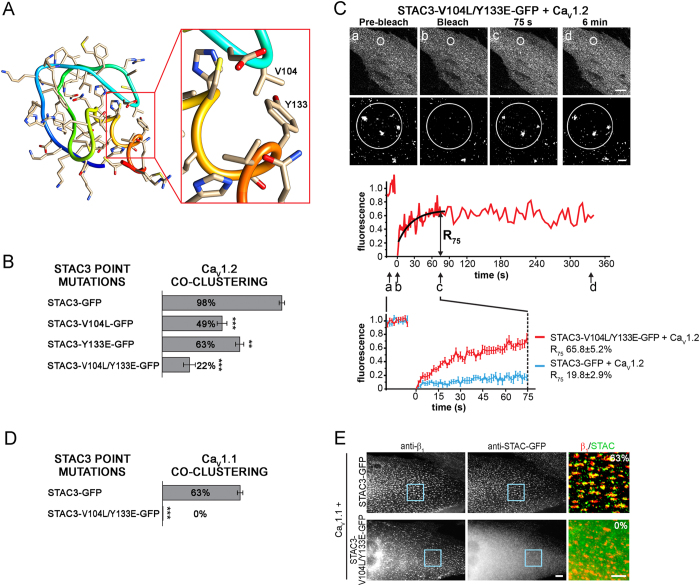
A binding pocket of STAC3 containing residues V104 and Y133 is critical for its association with L-type calcium channels in triads of dysgenic myotubes. (**A**) Homology structure model of the C1 domain of STAC3. The enlargement reveals the side chains of V104 and Y133 protruding into a pocket of the C1 domain. (**B**) V104 and Y133 were mutated individually and together to the corresponding residues of STAC2. STAC3-V104L and STAC3-Y133E showed reduced co-clustering with Ca_V_1.2 (46.7 ± 4.0% and 63.3 ± 3.3%, respectively), while the combined mutant STAC3-V104L/Y133E showed a further co-clustering reduction (22.2 ± 4.8%) compared to STAC3 (97.8 ± 2.2%; N = 3, n = 90). Anova F(3,8) = 71.4; P < 0.001 (P in the figures are for post-hoc analysis; ***P < 0.001; **P = 0.001). (**C**) FRAP analysis of STAC3-V104/Y133E-GFP co-expressed with Ca_V_1.2 shows substantial recovery of fluorescence. Average recovery curves reveal an approximately threefold higher recovery rate of STAC3-V104/Y133E-GFP compared to that of STAC3-GFP (mean ± s.e., N = 3, n = 18, Student’s t-test, P < 0.001). Upper scale bar: 10 μm. Lower scale bar: 1 μm. (**D**,**E**) When co-expressed with Ca_V_1.1, the double STAC3-V104L/Y133E mutant failed to colocalize in clusters, while the wild-type STAC3 colocalized with Ca_V_1.1 in 63.3 ± 1.9% of the transfected myotubes (N = 3, n = 90). Student’s t-test, P > 0.001. Color overlay: 4X of blue rectangle. Scale bars: 10 μm and 5 μm.

To examine the position of this critical V104 residue in the tertiary structure of STAC3 and to identify further candidate residues potentially involved in Ca_V_1.2 binding, we generated a protein structure homology model of the STAC3 C1 domain based on the available structures of C1 domains and using SWISS-MODEL^14^ (Fig. 7A). As expected for a residue involved in protein-protein interactions, V104 is exposed at the surface in a domain resembling a binding pocket. Importantly, within this pocket V104 is in close proximity to the side chain of residue Y133. If indeed this is the binding pocket of STAC3 involved in the association with Ca_V_1.2, also mutating the second residue in this pocket (Y133) would be expected to interfere with Ca_V_1.2 co-clustering. To test this hypothesis, we generated a STAC3 mutant in which Y133 was mutated to the corresponding residue in STAC2 (STAC3-Y133E-GFP) as well as a double point mutant in which both V014 and Y133 were mutated (STAC3-V104L/Y133E-GFP) (Fig. 7B). As hypothesized, when co-expressed with Ca_V_1.2, STAC3-Y133E-GFP showed a significantly reduced co-clustering compared to STAC3-GFP (respectively, 63.3 ± 3.3% and 97.8 ± 2.2%). The extent of reduction was similar to that of STAC3-V104L-GFP (48.9 ± 4.0%). Most importantly, when both V104 and Y133 were mutated (STAC3-V104L/Y133E-GFP) co-clustering with Ca_V_1.2 was even further diminished (22.2 ± 4.8%), consistent with a critical and synergistic function of the two residues in binding Ca_V_1 channels (Fig. 7B). FRAP analysis showed that, compared to the wild type STAC3-GFP (Fig. 3B), STAC3-V104L/Y133E-GFP had a dramatically increased fluorescence recovery within Ca_V_1.2 clusters (Fig. 7C). The R_75_ of STAC3-V104L/Y133E-GFP was 3-folds higher (R_75_, 65.8 ± 5.2%) than that of STAC3-GFP (R_75_, 19.8 ± 2.9%) and after 6 minutes its fluorescent clusters substantially recovered, unlike the wildtype STAC3-GFP (Fig. 1B). Thus, mutating V104 and Y133 in the C1 domain of STAC3 decreases the stability of the STAC3-Ca_V_1.2 interaction, corroborating the central role of this binding pocket in the stable association of STAC3 with Ca_V_1 channels.

In order to verify that residues V104 and Y133 of STAC3 are crucial also for the interaction with the skeletal muscle Ca_V_1.1 channel, we co-expressed the STAC3 double mutant and Ca_V_1.1 in dysgenic myotubes (Fig. 7D,E). While wildtype STAC3-GFP co-clusters with Ca_V_1.1 in approximately two thirds of the myotubes (63.3 ± 1.9%, see Fig. 1A), the STAC3-V104L/Y133E-GFP double mutant entirely failed to co-cluster but showed a diffuse localization in all of the observed myotubes (0.0 ± 0.0%). The complete failure of STAC3-V104L/Y133E-GFP incorporation into the triads highlights the crucial role of this binding pocket in the assembly of the native Ca_V_1.1-STAC3 complex, but also precludes FRAP analysis of the native complex stability. Thus, we can conclude that residues V104 and Y133 in the C1 domain of STAC3 are essential for the native interaction between STAC3 and Ca_V_1.1 in skeletal muscle triads.

## Discussion

The incorporation of STAC3-GFP into skeletal muscle Ca_V_1.1 complexes, the specificity of the STAC3-GFP/Ca_V_1.1 interaction among the members of the STAC family, and its fluorescence recovery rate similar to that of the Ca_V_β_1a_ subunit, identify STAC3 as an integral component of the skeletal muscle EC coupling complex. Co-immunoprecipitation and mass spectrometry analyses in zebrafish myotubes previously identified as interaction partners of STAC3 the RyR1, Ca_V_1.1 and the Ca_V_ auxiliary subunits^5^. However recruitment of heterologously expressed STAC3-GFP (Fig. 1A) or STAC3-YFP into triads failed in the absence of Ca_V_1.1 in dysgenic myotubes, but not in the absence of RyR1 in dyspedic myotubes^7^, suggesting that the putative interaction with RyR1 is too weak to recruit heterologously expressed STAC3-GFP into triads lacking Ca_V_1.1. Here we restored STAC3-GFP triad targeting in dysgenic myotubes by expressing Ca_V_1 subunits, and thus demonstrate that Ca_V_1.1 is the main interaction partner of STAC3 in the skeletal muscle EC coupling complex.

The extent to which Ca_V_1 subunits recruited STAC3 into triads is comparable to that of Ca_V_β subunits (Fig. 1B, ref. 9). Moreover Ca_V_1.1, β_1a_ and STAC3 all showed the same slow FRAP rates in the native environment of triads (Fig. 1C, ref. 9), indicating that STAC3 is a stable and integral part of the triadic DHPR complex, and thus could be considered to be a skeletal muscle-specific auxiliary Ca_V_1.1 subunit. The specific role of STAC3 in the skeletal muscle EC coupling apparatus is further supported by the observed isoform-specificity of the interaction of Ca_V_1.1 with STAC3. Ca_V_1.1 specifically recruited STAC3, but not STAC1 or STAC2 into triads of dysgenic myotubes (Fig. 4C). In contrast, the widely expressed Ca_V_1.2 isoform to different extents recruited all three STAC paralogs into the triads, although at greatly different extents (Fig. 4C).

The stability of STAC3-GFP in the Ca_V_1.1 complex also supports the notion that in skeletal muscle STAC3 does not merely function as a chaperone of Ca_V_1.1^7^, but that its permanent presence in the triads may be required for sustaining the function of the EC coupling apparatus. While at present the exact role of STAC3 in skeletal muscle EC coupling is still elusive, this scaffold protein either may be involved in maintaining the unique tetradic organization of the Ca_V_1 complex opposite the RyR1, or in the functional transmission of the EC coupling signal from the Ca_V_1 voltage sensor to the RyR1 release channel. Within the STAC3 protein the first SH3 domain has been implicated in EC coupling function because mutation of a conserved SH3 domain residue (W > S) causes reduced EC coupling in NAM^5^. Our co-clustering analyses revealed that this NAM mutation reduced the recruitment of STAC3 specifically into skeletal muscle Ca_V_1.1 channel complexes (Fig. 2), but not into triads reconstituted with Ca_V_1.2 (data not shown). This finding is in line with the importance of STAC3 for the membrane targeting of Ca_V_1.1 in heterologous cells, whereas membrane targeting of Ca_V_1.2 does not require STAC3^7^. Surprisingly however, the NAM mutation did not reduce the stability of STAC3 within the Ca_V_1.1 complex (Fig. 2). Therefore the first SH3 domain of STAC3 may be of importance for Ca_V_1.1 membrane targeting or for establishing functional interactions in skeletal muscle triads but does not determine the structural association of STAC3 and Ca_V_1.1. Consistent with our observation, Polster, *et al*.^8^ recently reported that the NAM mutation of STAC3 has minor effects on membrane expression of Ca_V_1.1 or its current properties in mouse myotubes, but causes a very large reduction in EC coupling.

By systematically testing the potential of multiple STAC2/STAC3 chimeras to incorporate in the DHPR complex in dysgenic myotubes, our gain-of-function study identifies the C1 domain of STAC3 as the putative Ca_V_1 binding site (Fig. 5). Although C1 domains were originally identified as lipid binding domains, increasing evidence suggests that C1 domains can also function in protein-protein interaction. C1 domains of β2-chimaerin and several PKC isoforms have been shown to bind a diverse collection of proteins^15^,^16^,^17^,^18^. Our present findings add the adaptor protein STAC3 and voltage-gated calcium channels to this growing list of protein partners interacting through a C1 domain. Within the C1 domain our mutagenesis analysis revealed the importance of residues V104 and Y133, which synergistically contribute to Ca_V_1 binding. Homology structure modeling of the STAC3 C1 domain showed that the side chains of these two residues protrude into a pocket, where an as of yet unidentified sequence of the Ca_V_1.1 channel may bind (Fig. 7A). Mutation of V104 and Y133 perturbed triad targeting of STAC3 in reconstituted dysgenic myotubes (Fig. 7B,D,E). The double mutation in the Ca_V_1 binding pocket of STAC3 decreased also its stability in the DHPR complex (Fig. 7C). These effects are reminiscent of the effects of β subunit mutations (M245A in β_2a_, M293A in β_1a_). These mutations decrease the affinity for Ca_V_α_1_ subunits^19^, and when expressed in dysgenic myotubes they reduced triad targeting of β_1a_ and increased its mobility within the DHPR complex by 3-fold^9^.

The identification of the N-terminal C1 domain of STAC3 as the Ca_V_1.1 binding site, and the fact that its mutation abolishes/weakens the stability of the STAC3-Ca_V_1 complex in the triads is in stark contrast to the effects of the NAM mutation in the SH3 domain. This indicates that the C1 and the SH3 protein-protein interaction domains determine the dual functions of STAC3 in Ca_V_1.1 binding and EC coupling, respectively. Whether the SH3 domain is important for Ca_V_1.1 targeting to the triads or whether it engages in functional low affinity interactions with the RyR1 or other triad proteins remains to be shown in future studies.

## Methods

### Cloning procedures

All plasmids are based on the pcDNA3 backbone and expression is under the control of a CMV promoter. Cloning procedures were previously described for pc-Ca_V_1.1 (NM_001101720)^20^.

*pc-Ca*_*V*_*1.2* (X15539). The coding sequence of rabbit cardiac Ca_V_1.2 was isolated from GFP-α_1C_^21^ with KpnI and Hind III and inserted in the corresponding sites of pc-Ca_V_1.1.

*pc-STAC1-GFP, pc-STAC2-GFP and pc-STAC3-GFP*. The STAC proteins were cloned with a GFP tag, to allow us to use the same antibody combination for the analysis of STACs co-clustering with Ca_V_1.1 and with Ca_V_1.2. The GFP-tag was placed at the C-terminal end of the proteins, since STAC3-eGFP and STAC3-YFP have been shown to promote functional EC coupling in reconstituted STAC3 KO zebrafish myotubes^5^ and Ca_V_1.1 membrane targeting in non-muscle cells^7^. The coding sequences of mouse STAC1 (NM_016853) and STAC2 (NM_146028) were isolated from mouse cortex cDNA, while STAC3 (NM_177707) was isolated from mouse soleus cDNA. Briefly, for each construct, a forward primer with a linker introducing a KpnI site upstream of the start codon and a reverse primer with a linker introducing a BamHI site after the stop codon were designed. The isolated STAC coding sequences were then digested with KpnI and BamHI and inserted in the corresponding sites of pc-β_1a_-GFP^20^, yielding pc-STAC1-GFP, pc-STAC2-GFP and pc-STAC3-GFP, where the STAC proteins and GFP are separated by a three amino acids linker (MDP).

The description for the cloning procedures of all the STAC chimeras and point mutations can be found in the Supplementary Methods.

### Dysgenic myotubes culture and transfection

Myotubes of the homozygous dysgenic (*mdg*/*mdg*) cell line GLT were cultured as previously described^22^. Cells grown on carbon and gelatin coated coverslips (or on plastic for WB analysis) were transiently transfected with the plasmids of interest 4 days after plating using FuGeneHD transfection reagent (Promega), according to the manufacturer’s instructions. A total of 0.5 μg of each plasmid DNA was used per 35 mm culture dish; for 60 mm dishes the amount was doubled; for 100 mm dishes 3.0 μg DNA were used.

### Immunocytochemistry and image processing

Dysgenic myotubes grown on coverslips were fixed in 4% paraformaldehye/4% sucrose in PBS at room temperature for 20 min and incubated in 5% normal goat serum in PBS containing 0.2% bovine serum albumin (BSA) and 0.2% Triton X-100 (PBS/BSA/Triton) for 30 min. The primary antibodies rabbit serum anti-GFP (1:10,000; Invitrogen), mouse monoclonal anti-RyR (1:1000, cl.34 C, Alexis Biochemicals), mouse monoclonal anti-β_1_ (1:2000, cl. N7/18, NeuroMab, University of California–Davis/National Institutes of Health NeuroMab Facility), mouse monoclonal anti Ca_V_1.1 (1:4000, cl. 1 A, Thermo Scientific), rabbit polyclonal anti Ca_V_1.2 (1:2000, Sigma) and mouse monoclonal anti-GFP (1:2000, cl. 270F3, Synaptic Systems), were applied in PBS/BSA/Triton either for 4 h at room temperature (RT) or overnight at 4 °C. The samples were then washed in PBS/BSA/Triton X and stained with goat anti-rabbit Alexa Fluor 488/594 and goat anti-mouse Alexa Fluor 488/594 (1:4000; Invitrogen) for 1 h at RT. After staining, coverslips were washed and mounted in Vectashield (Vector laboratories) to retard photo bleaching. Preparations were analyzed on an AxioImager microscope (Carl Zeiss) using 63 × 1.4 NA objective. Fourteen-bit images were recorded with a cooled CCD camera (SPOT; Diagnostic Instruments) and Metaview image-processing software (Universal Imaging). Figures were arranged in Adobe Photoshop CS6 and, where necessary, linear adjustments were performed to correct black level and contrast.

### STAC co-clustering analysis

Semi-quantative analysis of STAC co-clustering was performed by systematically screening for myotubes with more than 4 nuclei and clustered Ca_V_β_1_ in the red channel (mouse anti-β_1_), which also expressed STAC-GFP constructs, as seen in the green channel (rabbit anti-GFP). For each such myotube pictures were taken in the red and green channel. Later the images were analyzed and each myotube was classified as STAC co-clustered or not. This binary classification, widely used^9^,^23^,^24^,^25^,^26^, reliably assesses the ability of a certain protein to localize in the triads independently of cell to cell variations in protein expression. For each condition at least three separate experiments were analyzed. Results are expressed as mean ± s.e. All data were organized in MS Excel and analyzed using Student’s *t*-test or ANOVA with Tukey post-hoc analysis in SPSS statistical software (SPSS Inc., Chicago IL, USA).

### FRAP experiments and analysis

FRAP was performed as previously described^9^. Briefly, 9 DIV transfected GLT myotubes were imaged in physiological Tyrode solution containing (in mM): 130 NaCl, 2.5 KCl, 2 CaCl2, 2 MgCl2, 10 HEPES, 30 glucose using a SP-5 confocal microscope (Leica Microsystems) equipped with a 63×, 1.4 NA water-immersion lens at 37 °C in an incubation chamber (EMBLEM). Fluorescence was excited using the 488 nm line of the argon laser and recorded at a bandwidth of 500–550 nm. Images were acquired at 0.67 Hz in the pre-bleach, bleach and post-bleach phase (respectively 10, 3, and 50 frames). For extended observation, additional 54 frames were acquired at a 5 s interval. For imaging in the pre-bleach and post-bleach phases the laser was set to 15–20% of the initially adjusted laser power (70%). A circular 6 μm diameter ROI was photobleached by scanning with the 488 nm line of argon laser at 100% intensity. Inside the bleached region, three 1 μm diameter ROIs were placed each over a cluster and three in the cluster-free regions. The average fluorescence of the cluster-free regions was set as background. The average fluorescence of the three ROIs on the clusters was background-subtracted and corrected for the overall bleaching in each time frame. Then the average fluorescence of the clusters was normalized so that the pre-bleach intensity was set to 1 and the first frame after photobleaching to 0 and plotted as function of time. The analysis of fluorescence was performed using LAS AF software (Leica Microsystems). Recovery curves were fitted with a monoexponential function with pClamp software (version 8.0, Molecular Devices) and the value of the fitted curve at 75 s after bleaching was chosen to calculate the mean rate of fluorescence recovery (R_75_). Results are expressed as mean ± s.e. All data were organized in MS Excel and statistical differences were determined by Student’s *t* test.

### Homology modeling

The PDB file of the C1 domain of STAC3 protein was generated by Swiss-Model server (http://www.expasy.org/swissmod/SWISS-MODEL.html). The model was then analyzed and exported from UCSF Chimera^27^.

### Western blot analysis

DIV 9–10 GLTs expressing STAC1-GFP, STAC2-GFP, STAC2-GFP and STAC3-NAM-GFP with or without Ca_V_1.1 were scraped in RIPA buffer (50 mM TRIS-HCl, pH 8; 150 mM NaCl_2_; 10 mM NaF; 0.5 mM EDTA; 0.10% SDS; 10% glycerol; 1% igepal; 1x Protease Inhibitor Complete cocktail (Roche)). The lysates were then purified by centrifugation (4,000 *g*, 10 min, 4 °C). Protein concentrations were determined using a BCA assay (Thermo Scientific) according to manufacturer instructions. Twenty micrograms of protein were separated by SDS-PAGE (4–12%) at 196 V and 40 mA for 60 min and transferred to a PVDF membrane at 25 V and 100 mA for 3 h at 4 °C with a semidry-blotting system (Roth). The blot was incubated with rabbit anti-GFP (1:10,000; Invitrogen) and mouse anti-GAPDH (1:100,000; Santa Cruz Biotechnology) antibodies overnight at 4 °C and successively with HRP-conjugated secondary antibody (1:5000; Pierce) for 1 h at room temperature. The chemiluminescent signal was detected with ECL Supersignal West Pico kit (Thermo Scientific) and visualized with ImageQuant LAS 4000.

## Additional Information

**How to cite this article**: Campiglio, M. and Flucher, B. E. STAC3 stably interacts through its C1 domain with Ca_V_1.1 in skeletal muscle triads. *Sci. Rep.* **7**, 41003; doi: 10.1038/srep41003 (2017).

**Publisher's note:** Springer Nature remains neutral with regard to jurisdictional claims in published maps and institutional affiliations.

## Supplementary Material

Supplementary Information
