# Supplementary material for: STAC3 stably interacts through its C1 domain with CaV1.1 in skeletal muscle triads
Source: Sci Rep. 2017 Jan 23;7:41003. doi: 10.1038/srep41003 (PMC5253670; doi:10.1038/srep41003)
Supplement: Supplementary Information [file srep41003-s1.pdf]

Supplementary material

STAC3 stably interacts through its C1 domain with $Ca_V1.1$ in skeletal muscle triads

Marta Campiglio and Bernhard E. Flucher*

Department of Physiology and Medical Physics, Medical University of Innsbruck, 6020 Innsbruck, Austria.

Supplementary Methods

Detailed cloning procedures. *pc-STAC3-NAM-GFP* (W280S) was generated by SOE-PCR. Briefly, STAC3 was amplified by PCR using a reverse primer introducing the point mutation G>C at position nt 839. The PCR product was then inserted in the corresponding KpnI and TfiI of pc-STAC3-GFP.

pc-STAC2-SH3-2-GFP. The second SH3 domain of STAC3 was inserted into the respective position of the second SH3 domain of STAC2 by SOE-PCR as follows. A region (nt 463-1050) containing the first SH3 domain was isolated from pc-STAC2-GFP, while the second SH3 domain of STAC3 (nt 913-1080) was isolated from pc-STAC3-GFP. The two separate PCR products were fused in a SOE-PCR reaction. Finally, the SOE-PCR fragment and pc-STAC2-GFP were BspEI and BamHI digested and subsequently ligated.

pc-STAC2-SH3-1-GFP. The first SH3 domain of STAC3 was inserted into the respective position of the first SH3 domain of STAC2 by SOE-PCR as follows. Nucleotides 463-865 were isolated from pc-STAC2-GFP by PCR. The first SH3 domain of STAC3 (nt 727-906) was isolated from pc-STAC3-GFP, while the second SH3 domain of STAC2 (nt 1051-1224) was isolated from pc-STAC2-GFP. The three separate PCR products were fused in a SOE-PCR reaction. Finally, the SOE-PCR fragment and pc-STAC2-GFP were BspEI and BamHI digested and subsequently ligated.

pc-STAC2-SH3-1-2-GFP. The two SH3 domains of STAC3 were inserted into the respective position of the SH3 domains of STAC2 by SOE-PCR as follows. A region (nt 463-865) upstream the first SH3 domain was isolated from pc-STAC2-GFP, while the two SH3 domains of STAC3 were isolated from pc-STAC3-GFP (nt

727-1080). The two separate PCR products were fused in a SOE-PCR reaction. Finally, the SOE-PCR fragment and pc-STAC2-GFP were BspEI and BamHI digested and subsequently ligated.

pc-STAC3/STAC2-GFP. The N-terminal part of STAC3, containing the poly-E and the PKC-C1 domains, was inserted into the respective position of STAC2 by SOE-PCR as follows. The N-terminal region (nt 1-736) of STAC3 was isolated from pc-STAC3-GFP, while the C-terminal part of STAC2 (nt 865-1224), containing the two SH3 domains, was isolated from pc-STAC2-GFP. The two separate PCR products were fused in a SOE-PCR reaction. Finally, the SOE-PCR fragment and pc-STAC2-GFP were KpnI and BamHI digested and subsequently ligated.

pc-STAC2-polyE-GFP. The N-terminal part of STAC3, containing the poly-E region, was inserted into the respective position of STAC2 by SOE-PCR as follows. The N-terminal region (nt 1-274) of STAC3 was isolated from pc-STAC3-GFP, while the C-terminal part of STAC2 (nt 331-1224), containing the PKC-C1 and the two SH3 domains was isolated from pc-STAC2-GFP. The two separate PCR products were fused in a SOE-PCR reaction. Finally, the SOE-PCR fragment and pc-STAC2-GFP were KpnI and BamHI digested and subsequently ligated.

pc-STAC2-PKC-C1-GFP. The PKC-C1 domain of STAC3 was inserted into the respective position of the PKC-C1 domain of STAC2 by SOE-PCR as follows. The N-terminal region of STAC2 (nt 1-274) containing the poly-P region was isolated from pc-STAC2-GFP, while the PKC-C1 domain of STAC3 (nt 265-438) was isolated from pc-STAC3-GFP. The two SH3 domains of STAC2 were isolated from pc-STAC2-GFP (nt 505-1224).The three separate PCR products were fused in a SOE-PCR reaction. Finally, the SOE-PCR fragment and pc-STAC2-GFP were KpnI and BamHI digested and subsequently ligated.

pc-STAC3-PKC-C1-GFP. The PKC-C1 domain of STAC2 was inserted into the respective position of the PKC-C1 domain of STAC3 by SOE-PCR as follows. The N-terminal region of STAC3 (nt 1-264) containing the poly-E region was isolated from pc-STAC3-GFP, while the PKC-C1 domain of STAC2 (nt 331-504) was isolated from pc-STAC2-GFP. The two SH3 domains of STAC3 were isolated from pc-STAC3-GFP (nt 439-1080).The three separate PCR products were fused in a SOE-PCR reaction. Finally, the SOE-PCR fragment and pc-STAC2-GFP were KpnI and BamHI digested and subsequently ligated.

pc-STAC3-PKC-C1-A-GFP. The N-terminal part of the PKC-C1 domain of STAC2 was inserted into the respective position of the PKC-C1 domain of STAC3 by SOE-PCR as follows. The N-terminal region of STAC3 (nt 1-264) containing the poly-E region was isolated from pc-STAC3-GFP, while N-terminal part of the PKC-C1 domain of STAC2 (nt 331-397) was isolated from pc-STAC2-GFP. The C-terminal part of the PKC-C1 domain and the two SH3 domains of STAC3 were isolated from pc-STAC3-GFP (nt 331-1080).The three separate PCR products were fused in a SOE-PCR reaction. Finally, the SOE-PCR fragment and pc-STAC2-GFP were KpnI and BamHI digested and subsequently ligated.

pc-STAC3-PKC-C1-B-GFP. The C-terminal part of the PKC-C1 domain of STAC2 was inserted into the respective position of the PKC-C1 domain of STAC3 by SOE-PCR as follows. The N-terminal region of STAC3 (nt 1-330) containing the poly-E region and the first part of the PKC-C1 domain was isolated from pc-STAC3-GFP, while C-terminal part of the PKC-C1 domain of STAC2 (nt 398-504) was isolated from pc-STAC2-GFP. The two SH3 domains of STAC3 were isolated from pc-STAC3-GFP (nt 439-1080).The three separate PCR products were fused in a SOE-PCR reaction. Finally, the SOE-PCR fragment and pc-STAC2-GFP were KpnI and BamHI digested and subsequently ligated.

pc-GFP-PKC-C1. The PKC-C1 domain of STAC3 was amplified from pc-STAC3-GFP with primers introducing a SalI and an EcoRI site at the 5´and 3´termini, respectively. The PCR product was then digested with EcoRI and SalI and inserted in the corresponding sites of GFP-α_{1S} [1].

pc-STAC3-GFP point mutants (K98R, P99A, K100S, F101P, D103E, V104L, M108L, I126V, Y133E). Briefly the cDNA sequence of STAC3 (nt 1–1080) was PCR amplified in separate PCR reactions using pc-STAC3-GFP as template with overlapping primers mutating the amino acid of interest. The two separate PCR products were then used as templates for a PCR reaction with flanking primers to connect the nucleotide sequences. The resulting fragment was then KpnI/BamHI digested and ligated into the corresponding sites of pc-STAC3-GFP.

pc-STAC3-V104L/Y133E-GFP. The cDNA sequence of STAC3-V104L (nt 1–1080) was PCR amplified in separate PCR reactions using pc-STAC3-V104L-GFP as template with overlapping primers introducing the mutation T>G and C>A at positions 397 and 399. The two separate PCR products were then used as

templates for a PCR reaction with flanking primers to connect the nucleotide sequences. The resulting fragment was then KpnI/BamHI digested and ligated into the corresponding sites of pc-STAC3-GFP.

Sequence integrity of the all newly generated constructs was confirmed by sequencing (MWG Biotech, Martinsried, Germany).

Supplementary Figures

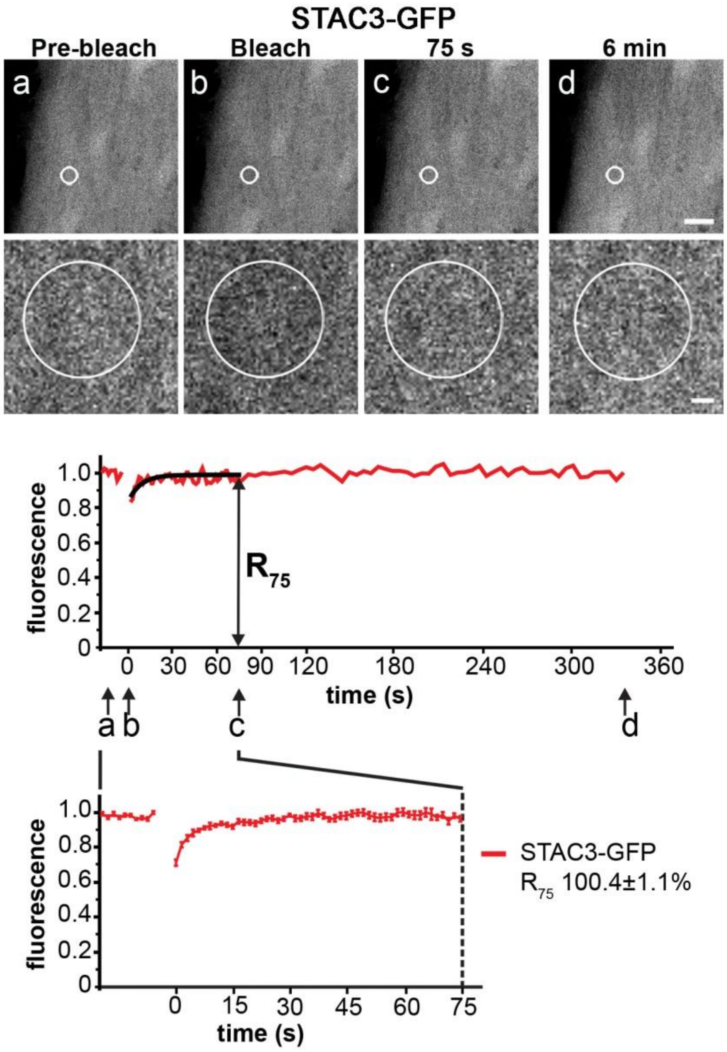

Fig. S1. FRAP analysis of STAC3-GFP in dysgenic myotubes. STAC3-GFP expressed without an $Ca_V\alpha_1$ subunit in dysgenic myotubes is diffusely distributed and its fluorescence recovers within few seconds after photobleaching (mean±SE, N=3 n=12), similar to $Ca_V\beta$ subunits [2] (upper bar, 10 μm; lower bar, 1μm).

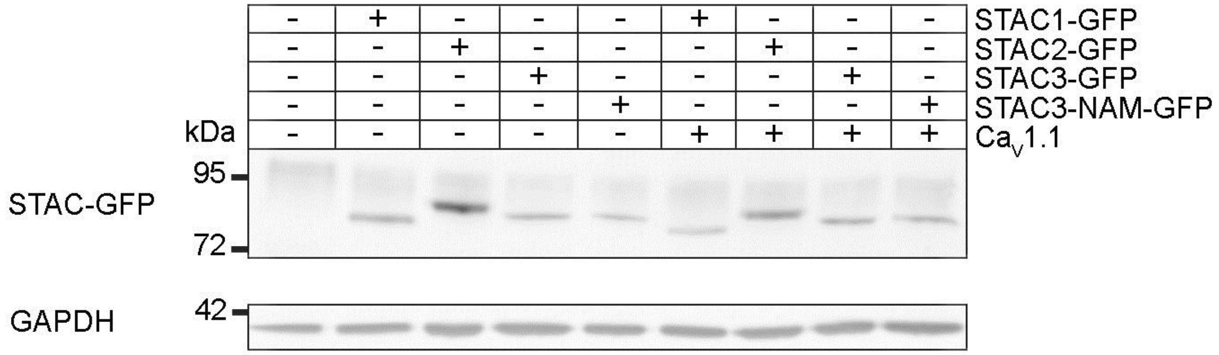

Fig. S2. STAC proteins expression levels in dysgenic myotubes are not correlated to their ability to co-cluster with Ca_V1.1. Western blot detection of STAC1-GFP, STAC2-GFP, STAC3-GFP, and STAC3-NAM-GFP, expressed with or without Ca_V1.1 in dysgenic myotubes, using the anti-GFP antibody. GAPDH was the loading control (N=3).

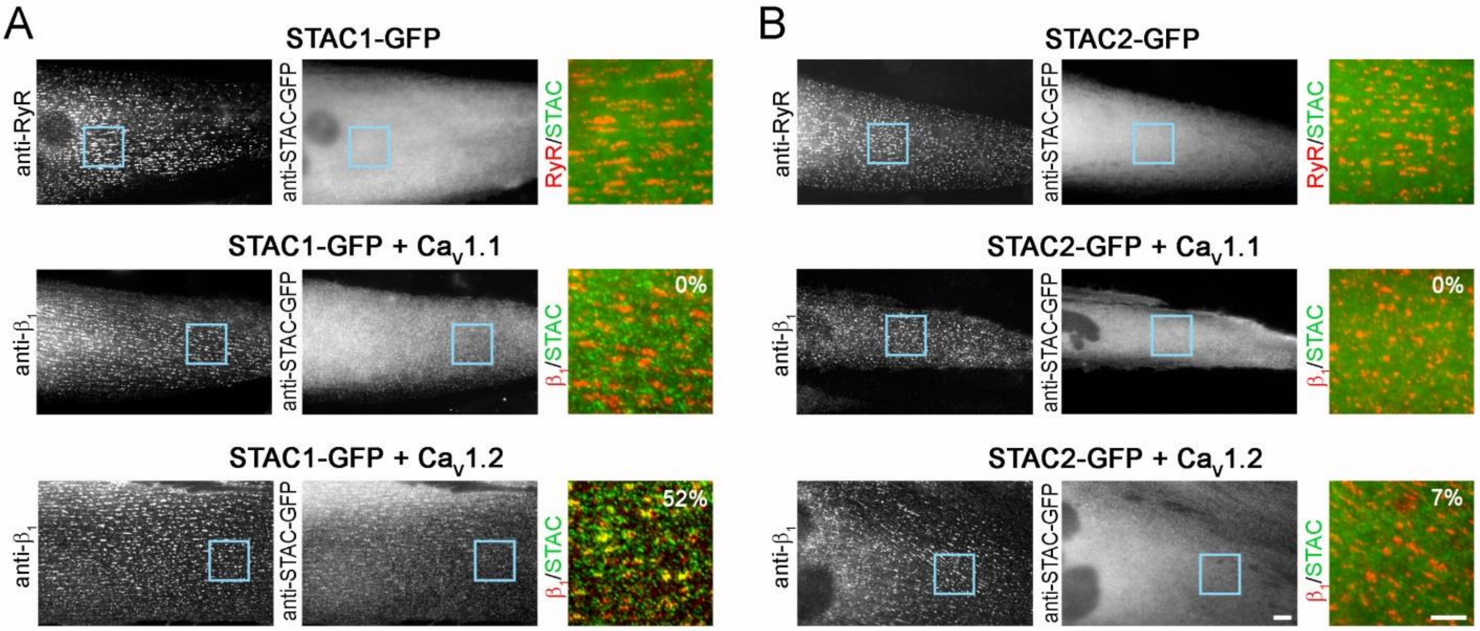

Fig. S3. STAC1 and STAC2 display different capacity of co-clustering with Ca$_V$1.1 and Ca$_V$1.2 calcium channels. (**A**) When expressed without a Ca$_V$1 subunit or when co-expressed with Ca$_V$1.1, GFP-labeled STAC1 homogenously distributes in the cytosol of dysgenic (Ca$_V$1.1$^{-/-}$) myotubes. However, when co-expressed with Ca$_V$1.2, STAC1 colocalizes with the calcium channel complex (here the DHPR β_{1a} subunit) in triad clusters of about half of the transfected myotubes. (**B**) GFP-labeled STAC2 shows a diffused cytoplasmic localization when expressed without a Ca$_V$1 subunit or co-expressed with either Ca$_V$1.1 or Ca$_V$1.2 (N=4, n=120). Color overlay: 4X of blue rectangle. Scale bars: 10 µm and 5 µm.

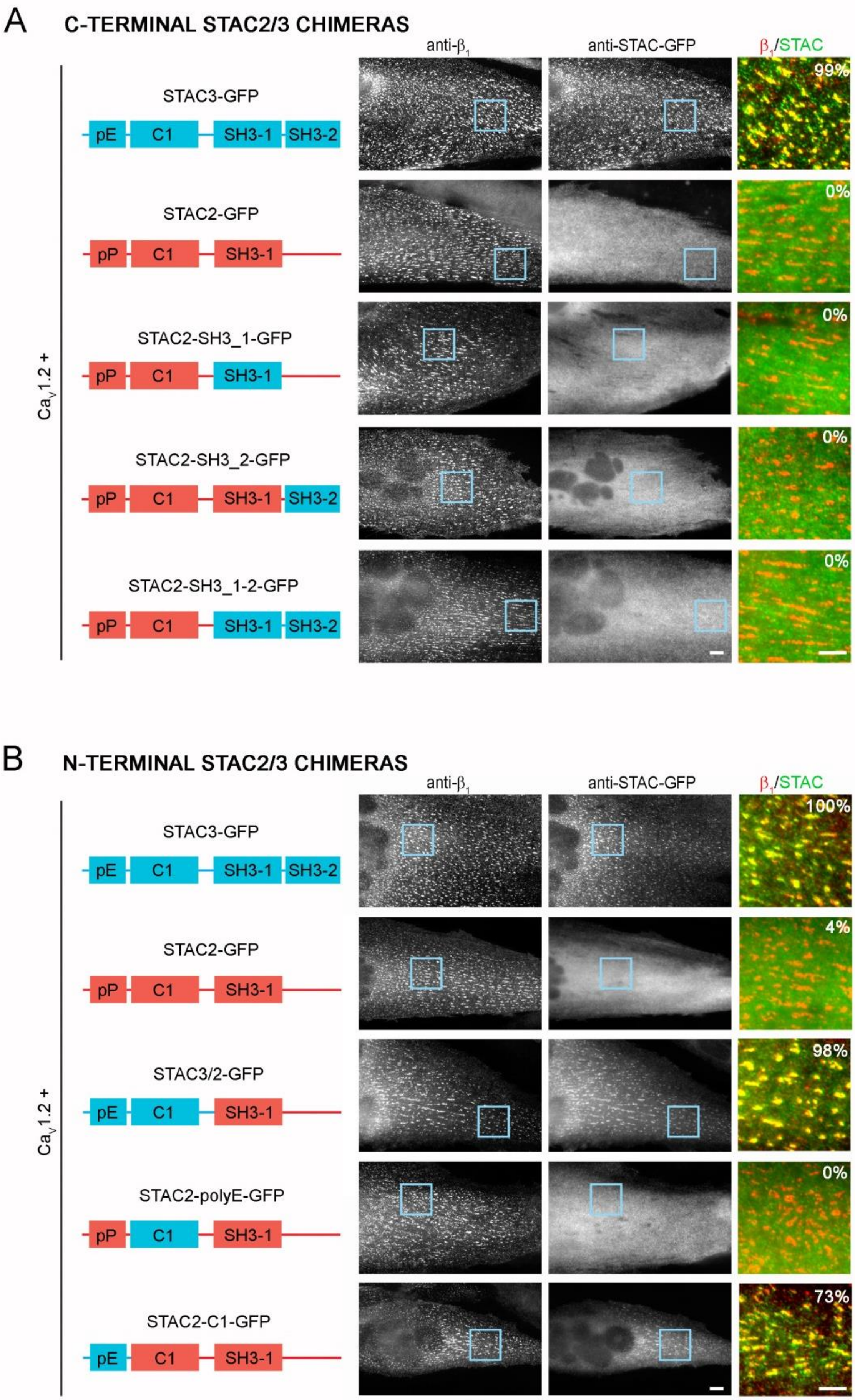

A
C-TERMINAL STAC2/3 CHIMERAS
anti-β1
anti-STAC-GFP
β1/STAC
CaV1.2 +
STAC3-GFP
pE C1 SH3-1 SH3-2
99%
STAC2-GFP
pP C1 SH3-1
0%
STAC2-SH3_1-GFP
pP C1 SH3-1
0%
STAC2-SH3_2-GFP
pP C1 SH3-1 SH3-2
0%
STAC2-SH3_1-2-GFP
pP C1 SH3-1 SH3-2
0%
B
N-TERMINAL STAC2/3 CHIMERAS
anti-β1
anti-STAC-GFP
β1/STAC
CaV1.2 +
STAC3-GFP
pE C1 SH3-1 SH3-2
100%
STAC2-GFP
pP C1 SH3-1
4%
STAC3/2-GFP
pE C1 SH3-1
98%
STAC2-polyE-GFP
pP C1 SH3-1
0%
STAC2-C1-GFP
pE C1 SH3-1
73%

Fig. S4. The C1 domain of STAC3 is the protein sequence critical for the interaction of STAC3 with Ca_V1.2. (**A**) The diffuse anti-STAC-GFP staining pattern of the C-terminal STAC2/3 chimeras indicates that none of the SH3 domains of STAC3 could confer to STAC2 the ability to colocalize with β_{1a} in the Ca_V1.2 complex (0.0±0.0%; N=3, n=90). (**B**) The substitution of the whole N-terminus (STAC3/2-GFP) or of the C1 domain of STAC2 for that of STAC3 (STAC2-C1-GFP) restored co-clustering of the chimera with Ca_V1.2 in the large majority of myotubes (98.3±1.0% and 73.3±4.9%, respectively; N=4, n=120). Color overlay: 4X of blue rectangle. Scale bars: 10 µm and 5 µm.

References

1 Grabner, M., Dirksen, R. T. & Beam, K. G. Tagging with green fluorescent protein reveals a distinct subcellular distribution of L-type and non-L-type Ca2+ channels expressed in dysgenic myotubes. *Proceedings of the National Academy of Sciences of the United States of America* **95**, 1903-1908 (1998).

2 Campiglio, M., Di Biase, V., Tuluc, P. & Flucher, B. E. Stable incorporation versus dynamic exchange of beta subunits in a native Ca2+ channel complex. *Journal of cell science* **126**, 2092-2101, doi:10.1242/jcs.jcs124537 (2013).
